# Functional morphology and efficiency of the antenna cleaner in *Camponotus rufifemur* ants

**DOI:** 10.1098/rsos.150129

**Published:** 2015-07-22

**Authors:** Alexander Hackmann, Henry Delacave, Adam Robinson, David Labonte, Walter Federle

**Affiliations:** 1 Department of Zoology, University of Cambridge, Downing Street, Cambridge CB2 3EJ, UK; 2 Warwick Medical School, University of Warwick, Coventry CV4 7AL, UK

**Keywords:** biomechanics, biomimetics, surface cleaning, insects, functional morphology

## Abstract

Contamination of body surfaces can negatively affect many physiological functions. Insects have evolved different adaptations for removing contamination, including surfaces that allow passive self-cleaning and structures for active cleaning. Here, we study the function of the antenna cleaner in *Camponotus rufifemur* ants, a clamp-like structure consisting of a notch on the basitarsus facing a spur on the tibia, both bearing cuticular ‘combs’ and ‘brushes’. The ants clamp one antenna tightly between notch and spur, pull it through, and subsequently clean the antenna cleaner itself with the mouthparts. We simulated cleaning strokes by moving notch or spur over antennae contaminated with fluorescent particles. The notch removed particles more efficiently than the spur, but both components eliminated more than 60% of the particles with the first stroke. Ablation of bristles, brush and comb strongly reduced the efficiency, indicating that they are essential for cleaning. To study how comb and brush remove particles of different sizes, we contaminated antennae of living ants, and anaesthetized them immediately after they had performed the first cleaning stroke. Different-sized beads were trapped in distinct zones of the notch, consistent with the gap widths between cuticular outgrowths. This suggests that the antenna cleaner operates like a series of sieves that remove the largest objects first, followed by smaller ones, down to the smallest particles that get caught by adhesion.

## 1. Introduction

The body surfaces of insects are exposed to a variety of microparticles, both man-made and natural, including biological particles (viruses, bacteria, spores and pollen), and inorganic matter such as mineral dust, salt and ash. Contamination by microorganisms, fungal spores and parasitoids can be life-threatening to insects [1,2] and can disrupt fundamental physiological functions such as the ability to climb [3–5], fly [6,7] or sense the environment [8,9]. Particles are deposited from the air, or transferred from surfaces touched by the insects. Natural contaminants range in size from nanometres to millimetres [10,11], but particles deposited on leaf and inert surfaces were found to have median diameters between 1 and 10 μm [12].

Insects have developed a variety of mechanisms to cope with contamination, including surfaces with self-cleaning properties [6,7,9] and active cleaning of body parts with specialized and complex cleaning structures [13–16]. Indeed, grooming represents one of the main activities in the life of insects [17]. Grooming is used not only to remove contaminants and pathogens but also to distribute cuticular hydrocarbons and antiseptic secretions on the body surface [8,18]. Insect grooming movements have been classified as ‘nibbling’, where cleaning is performed by the insect's mouthparts, ‘rubbing’, where an appendage sweeps back and forth in continuous contact over another body part, and ‘scraping’, which consists of unidirectional movements performed by the cleaning structure [19].

Insect cleaning systems typically include arrays of inclined hairs or projections that are moved across the surfaces to be cleaned [14]. These hairs are thought to help both with the mechanical removal by scraping or interdigitation as well as with the transport and concentration of particles [14]. Flexible setae are also known to provide strong adhesion in the footpads of many climbing animals [20,21], but so far the possible role of adhesion has not been considered for insect cleaning hairs. It is likely that not only the morphology of the hairs but also their spacing have important implications for their performance in the cleaning process. However, the existing knowledge of insect cleaning structures is based mainly on anatomical studies, and little is known about the efficiency and biomechanics of particle removal.

**Figure 1.**
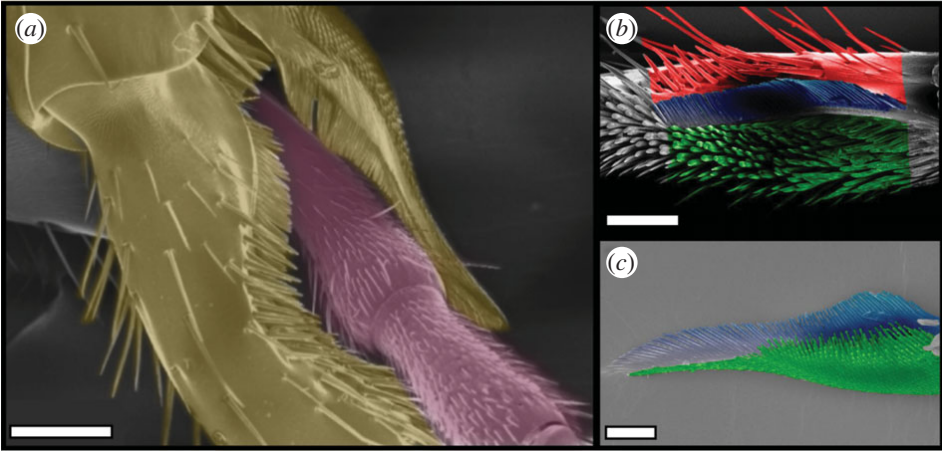
Antenna cleaner of *C. rufifemur* ants. (*a*) Scanning electron micrograph of the antenna clamped by the cleaner (view from the outer=posterior side). (*b*) Tarsal notch. (*c*) Tibial spur. Images in (*b*,*c*) are coloured to show the bristles (red), the comb (blue) and the brush (green). Scale bars, (*a*) 150 μm, (*b*) 100 μm and (*c*) 50 μm.

Here, we study the functional morphology of the ants' antenna cleaner. When ants clean their antennae, they lower their head slightly and clamp one antenna in a cleaning structure on the front leg of the same side, and pull the antenna through, while the clamp is held in contact. The cleaner is located at the tibia–tarsus joint and consists of a concave region of the basitarsus (notch) facing a flexible spur articulated at the end of the tibia. The spur joint lacks musculature and cannot be actively controlled [22,23]. However, the clamp can be opened and closed by changing the tibia–tarsus joint angle [24]. Both parts of the cleaning structure bear combs (i.e. rows of stiff, regularly spaced cuticular outgrowths), and brush-like structures (arrays of flexible setae, figure 1). Thus, the cleaning process involves both a macroscopic interaction between the two-part clamp and the antenna as well as a microscopic interaction between cleaning hairs and antennal sensilla. Similar antenna cleaners on front legs are present in many other groups of insects, and they consist either of multiple rows of projections as in ants, or just a single row as in some beetles [15,25]. Previous work noted the diverse morphologies of cleaning hairs in the hymenopteran antenna cleaner [26] but their function is still unknown.

In this article, we investigate (i) whether the hair arrays on notch and spur are required for efficient cleaning and (ii) how the morphology and spacing of the surface structures on the antenna cleaner contribute to the removal of different-sized particles from the antenna.

## 2. Material and methods

Experiments were carried out with a colony of *Camponotus rufifemur* ants collected from Brunei (Borneo). The ants were kept at 25°C on a 12 L : 12 D cycle and fed with honey-water and dead insects ad libitum. For behavioural observations, *C. rufifemur* workers were placed in a Petri dish and filmed for 2 min using a video camera. We also prepared close-up video recordings of the antenna pulled through the cleaning structure by mounting the antenna on a motorized translation stage and positioning the front leg appropriately by using two independent micromanipulators.

### 2.1 Morphometry of cleaning structures

The tibia and tarsus of front legs were severed from anaesthetized ants, air-dried and mounted on scanning electron microscope (SEM) stubs using double-sided carbon tape. Samples were sputter-coated at 65 mA for 15 s with a layer of gold (approx. 15 nm thick) using a Quorum Technologies K575X turbo-pump sputter coater (Laughton, UK). Images were taken using a LEO GEMINI 1530VP field emission gun scanning electron microscope (Thornwood, USA) with a beam voltage of 5 kV. Morphological characters were measured using ImageJ v. 1.46a [27]. The gap-size between cleaning hairs and between the hair-like antennal sensilla (including sensilla basiconica, chaetica and trichodea) was measured as the surface-to-surface distance to the nearest hair 5 μm below the tip, as the hairs have a tapered or conical shape. The sensilla base diameter was calculated as the average of five randomly selected sensilla per individual (*n*=10 ants).

### 2.2 Particle removal efficiency in simulated cleaning trials

To estimate the particle removal efficiency of the antenna cleaner, we replicated the cleaning movement of ants using a motorized set-up. One antenna of *C. rufifemur* was glued over its whole length to an insect pin using superglue (Loctite, Henkel, Hatfield, England), in order to prevent bending of the antenna, and the insect pin was attached to a micromanipulator. The whole tarsus of a front leg (for the notch) or only the spur were also attached to a stabilizing insect pin connected to a custom-built force transducer mounted on a three-dimensional motor positioning stage (M-126PD, C-843; Physik Instrumente, Karlsruhe, Germany). The force transducer consisted of a metal cantilever with 350 Ohm foil strain gauges (1-LY13–3/350; Vichay, Raleigh, NC, USA) mounted in a Wheatstone half-bridge configuration.

**Figure 2.**
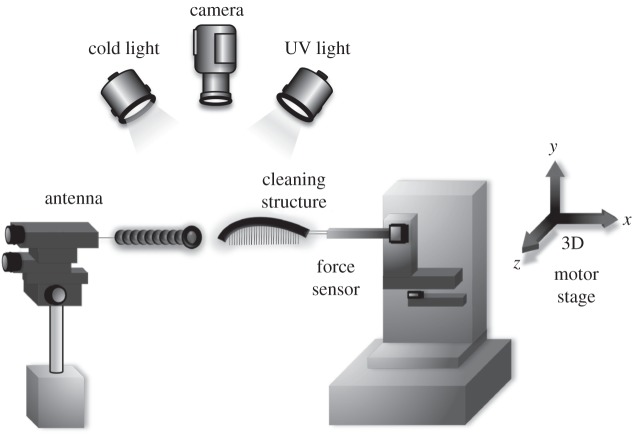
Set-up for performing simulated strokes with the ants' antenna cleaner.

Antennae were fully covered in yellow, UV-fluorescent polystyrene (PS) particles (3.5–4.5 μm diameter; Eventlights, Laakirchen, Austria) using a paintbrush. Following contamination, the cleaning structure (notch or spur) was brought into contact with the antenna with a normal force of 0.2 mN (we estimated this range of clamping forces in a preliminary study, which combined three-dimensional video recordings of grooming behaviour in living ants with measurements of the spur's clamping force in dependence of the tibia–tarsus angle; A.H. 2015, unpublished data), controlled via a force feedback mechanism implemented in a custom-made Labview program (National Instruments, Austin, TX, USA; figure 2). To simulate grooming, the axis of the tarsus or spur was positioned at right angle to the antenna, and the structure was tilted so that bristles, comb and brush were in contact with the antenna surface. Cleaning structures were then moved over the antennae at 1 mm s^−1^ over a distance of 3 mm, keeping the normal force constant at 0.2 mN. We conducted two consecutive movements of the cleaner over the antenna, as observed in natural cleaning (see Results). A digital camera (Nikon Coolpix 4500, Tokyo, Japan) mounted on a stereomicroscope (Leica Microsystems, Wetzlar, Germany) was used to record the antenna immediately after contamination and after each cleaning movement, using UV illumination (Omnilux CFL 25W, London, UK), where the fluorescent particles were visible with high contrast. As the antenna was sometimes slightly displaced by the cleaning movements, consecutive images of the surface of the antenna were aligned using GIMP2 (v. 2.8) and a 50×50 μm region of interest was cut out for images of all three treatments (contaminated, after first and second cleaning stroke). This region was analysed using a threshold algorithm in ImageJ to measure the degree of contamination. The cleaning efficiency of notch and spur was determined by measuring the area coverage of fluorescent particles before and after the cleaning strokes.

In order to investigate the contribution of the bristles, combs and brushes, we removed the hairs using a heated insect pin attached to the tip of a soldering iron. Simulated cleaning movements were performed with these ablated cleaning structures as described above.

The effect of consecutive cleaning strokes was statistically analysed using Page's non-parametric trend test [28], where the indices *L*_*m*,*n*_ indicate the sample size (*m*) and the number of conditions (*n*).

### 2.3 Removal of different-sized particles by the cleaning structure

In order to investigate which parts of the antenna cleaner contribute to the removal of different-sized particles, antennae of *C. rufifemur* were contaminated with PS beads of four different diameters (43, 25, 10 and 1 μm; Polybeads, Polysciences Inc., Eppelheim, Germany). Ants (*n*=7 workers per particle size) were briefly cooled at 5°C; they were then held with insect tweezers to pull their antennae through a suspension of the PS particles (1 μm: 2.63% solids in water; 10 μm: 2.05%; 25 μm: 2.61%; 43 μm: 2.54%). We placed the ants in a Petri dish and waited until they recovered from anaesthesia and started to groom their antennae. Immediately after the first grooming movement, i.e. before the cleaning structures were cleaned by the mouthparts, we anaesthetized the ant again by gently blowing CO_2_ into the Petri dish through a small hole in the lid. The antenna cleaner was then removed and prepared for SEM. We confirmed by prior light microscopy of the samples that no larger particles were knocked off during sputtering and imaging in the SEM. In order to analyse the function of the three distinct areas on the notch, i.e. bristles, comb and brush, we counted the number of particles trapped in each of these zones after one cleaning stroke. For the bristles, we included all particles that were located posterior to the comb. Particles can become deposited in this area as the antenna enters the cleaning structure from the posterior (bristle) side. For the comb area, every particle touching the comb was counted. We always viewed both sides of the comb by rotating the stubs in the SEM or reorienting specimens manually. For the brush area, all particles in contact with the cleaning hairs were counted. For the 1 μm particles in the brush, it was impossible to count total numbers. Therefore, particles were counted in an area of 50×50 μm, and the total number was calculated based on the overall size of the brush area. For each particle size, we calculated the percentage of particles found in each of the three areas of the notch by dividing the number of particles in one area by the number of particles in all areas. This approach was required to correct for the considerable differences in particle numbers between particle sizes. Data reported in the text are given as mean±s.d. unless indicated otherwise. Results are shown as boxplots, where the boxes, centre lines and whiskers show the interquartile range, median and 1.5× interquartile length, respectively. Statistical analyses were performed using SPSS (IBM, Armonk, USA) and R v. 3.1.2 (The R Foundation for Statistical Computing, Auckland, New Zealand).

## 3. Results

### 3.1 Cleaning movements

When cleaning the antenna, *C. rufifemur* ants pulled the flagellum (the part of the antenna beyond the antennal scape) through the cleaning device on the ipsilateral foreleg while holding it clamped between tarsal notch and tibial spur (see electronic supplementary material, video S1). During this process, the flagellomeres move from the posterior to the anterior side of the foreleg. The ants pulled the antenna mostly twice through the antenna cleaner before the cleaner itself was cleaned with the mouthparts (once: 33% of all cleaning movements; twice: 58%; more than two times: 9%; *n*=88 cleaning movements from 10 ants).

Microscopy images of the antenna positioned in the cleaning structure (figure 1*a*) and close-up video recordings of the pulling movements show that the bristles and combs are oriented obliquely to the antennal surface during cleaning (see electronic supplementary material, video S2).

### 3.2 Morphometry of cleaning structures

The tarsal notch and the tibial spur of the antenna cleaner in *C. rufifemur* ants bear different types of hairs that are arranged in distinct regions (figure 1). Both notch and spur contain a regular ‘comb’ on the posterior side and a ‘brush’ array of flexible setae on the anterior side; the notch also has a ‘bristle’ region with thick conical hairs posterior to the comb (figure 1).

**Figure 3.**
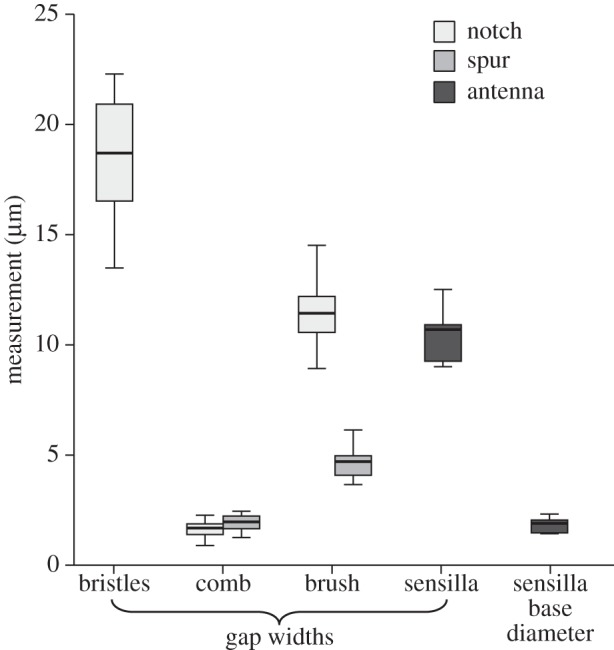
Gap widths between cleaning hairs on the tarsal notch and the tibial spur, in comparison with the spacing and base diameter of the sensilla on the antennae.

Measurements on the tarsal notch showed that the gaps between hairs/bristles were widest for the bristles, narrowest for the comb and intermediate for the brush. All pairwise comparisons for gap width were significant (Bonferroni–Holm corrected paired *t*-tests: *p*<0.01, figure 3).

On the tibial spur, the comb also showed narrower gaps than the brush (five measurements each from *n*=10 ants; *t*_9_=10.9, *p*<0.001). A comparison between the structures on spur and notch showed that there was a very similar gap width for the combs (*t*_9_=1.6, *p*=0.967), but not for the brushes where the gaps between adjacent setae were wider on the notch (*t*_9_=12.2, *p*<0.001). The gap widths of the combs on spur and notch were similar to the base width of the antennal sensilla (notch: *t*_9_=1.2, *p*=0.48; spur: *t*_9_=0.58, *p*=0.57).

### 3.3 Cleaning efficiency of the antenna cleaner

**Figure 4.**
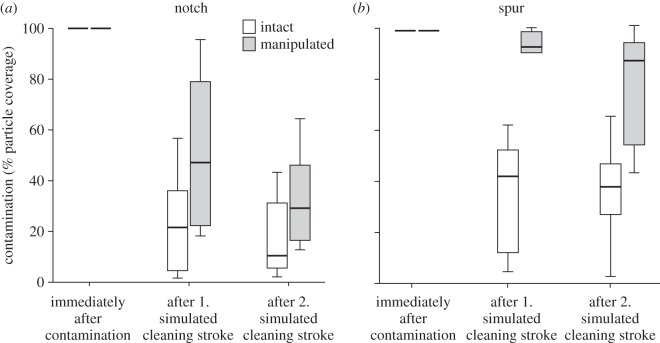
Cleaning efficiency of intact and manipulated tarsal notch (*a*) and tibial spur (*b*). For the manipulation, bristles, comb and brush setae were ablated.

Particles were efficiently removed when an intact tarsal notch was dragged across a contaminated antenna (figure 4; Page's trend test: *n*=10; *L*_10,3_=136.5, *p*<0.001). Two cleaning strokes of the tarsal notch reduced the particle coverage by 84% (median). When bristles, comb and brush setae were ablated from the tarsal notch, particles were still removed (*n*=10; *L*_10,3_=137; *p*<0.001), but less efficiently (71% median) than for the intact notch (Mann–Whitney *U*-test, comparison between intact and manipulated notch after two cleaning strokes: *W*=73; *n*=10; *p*=0.016).

Similar to the tarsal notch, the tibial spur cleaned the contaminated antenna significantly with two cleaning strokes (*n*=10; *L*_10,3_=135; *p*<0.001), and removed 64% (median) of the particles. As for the notch, the cleaning performance of the manipulated spur was still significant (*n*=10; *L*_10,3_=134; *p*<0.01), but much less efficient (12% median) than the intact spur (Mann–Whitney *U*-test: *W*=60; *n*=10; *p*<0.001).

A comparison between intact notch and spur showed a significantly higher cleaning efficiency of the notch after the second stroke (*W*=75; *n*=10; *p*=0.0233).

### 3.4 Destination of different-sized particles on the cleaning structure

**Figure 5.**
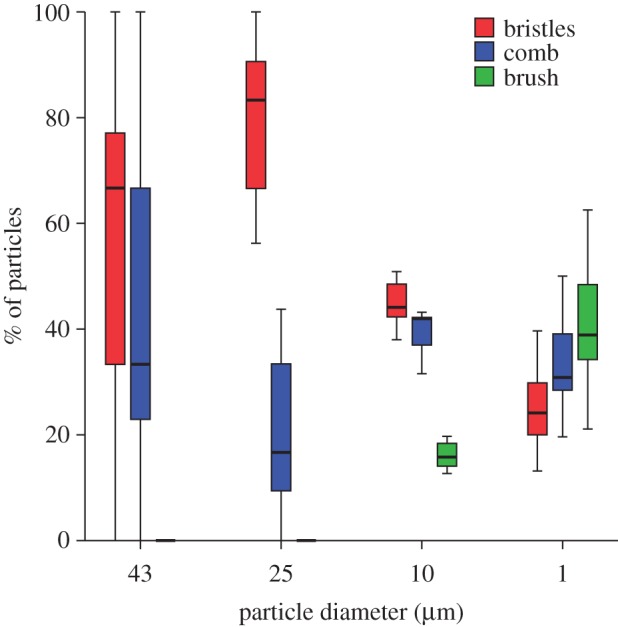
Location of particles of different size on the tarsal notch after a single cleaning stroke, given for each particle size as the percentage found on bristles, comb and brush. For each particle size, *n*=7 cleaning structures of different ants.

The analysis of the distribution of different-sized particles in each area of the notch (bristles, comb and brush) revealed that the larger (43 μm, 25 μm) particles were found almost exclusively in the bristles and the comb, whereas the 10 μm particles could also be found in the brush. The smallest particles (1 μm) were caught mainly in the brush (figure 5). Particle size had a significant effect on particle distribution in the bristles and the brush, but not in the comb (Jonckheere trend tests, four levels, *n*=28, corrected for multiple testing; bristles: JT=218.5, *p*=0.0068; comb: JT=141.5, *p*=0.8216; brush: JT=22, *p*<0.001). The large variation of the percentages measured for the 43 μm particles can be attributed to the small number of particles present.

## 4. Discussion

The maintenance of clean and functional body surfaces is of vital importance for insects [8,9]. Most Hymenoptera possess a two-part cleaning structure on their front legs that serves to clamp and clean their antennae [29]. By simulating cleaning strokes of both parts of the antennal cleaner on contaminated antennae in *C. rufifemur* ants, we found that both the tarsal notch and the tibial spur removed particles efficiently from the surface of the antenna.

Our results confirm that the cleaning process depends on both the macroscopic contact between cleaning structure and contaminated antenna [24,26], and on the microscopic interaction between cleaning hairs, antennal sensilla and contaminating particles. Removal of the bristles, comb and brush structures from notch and spur showed that some particles were removed even by the ablated structures, but the cuticular outgrowths strongly increased the cleaning efficiency.

### 4.1 Microscopic combs and brushes act as filters for particles of different size

The SEM analysis of the particle distribution after a cleaning stroke showed that the ordered arrangement of the three different hair arrays (bristles, comb and brush) on the tarsal notch and their specific morphology help ants to remove surface contaminants of different sizes.

After the cleaning stroke, the largest (43 μm) particles were found either outside the bristles or between bristles and comb. The gaps between the bristles are too small to allow the passage of the largest particles (median gap width 18.7 μm) so that these are scraped off the antenna. The 25 μm particles are still too large to pass through and also get scraped off the antenna, but they are small enough to enter and settle in between the bristles. As the 10 μm particles are sufficiently small to pass through the gaps between the bristles, they reach the comb where they are picked up by the more tightly set comb teeth (median gap width 1.7 μm). The oblique orientation helps to lift the particles off the antennal surface; they then remain attached to the hairs of the cleaning structure. These findings confirm earlier ideas about the function of cleaning hairs derived from their morphology [14].

**Figure 6.**
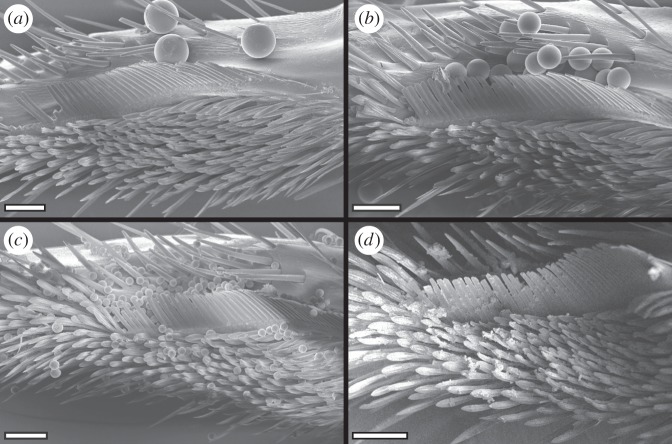
Tarsal notch after a single cleaning stroke over an antenna contaminated with particles of different sizes. (*a*) 43 μm, (*b*) 25 μm, (*c*) 10 μm, (*d*) 1 μm particles. (*a*–*d*) Scale bars, 50 μm.

**Figure 7.**
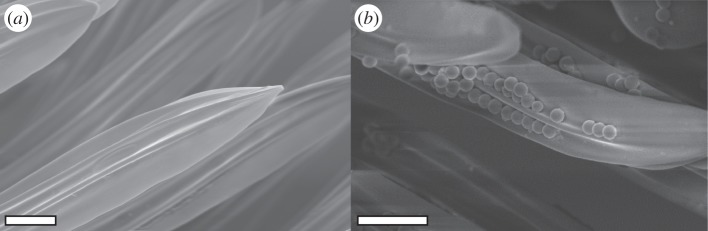
Brush setae in the cleaning structure in *C. rufifemur*. (*a*) Cuticular ridges. (*b*) 1 μm PS particles adhering to setae. (*a*,*b*) Scale bars, 5 μm.

Finally, the smallest (1 μm) particles were mainly captured by the brush (figure 6). Here, the gaps between the brush hairs are much larger than the particles, and the majority of particles must therefore be picked up from the antenna by a different mechanism. The flattened brush hairs are flexible, which may help them to come into intimate contact with a large number of particles on the antennal surface. The setae can easily bend and adapt to the shape of the convex antenna (figure 7*a*). In addition, the ridged surface structure of the setae may cause small particles to adhere within the surface ridges (figure 7*b*). Moreover, the brush zone is relatively extended (along the axis of the antenna) compared with the comb and bristles, thereby increasing the number of brush hairs interacting with the antenna (figure 1). Thus, the brush setae are likely to be similar in function to adhesive setae used for climbing [20].

Particles are removed if their adhesion to the brush setae outweighs their adhesion on the antennal surface. This may occur by several mechanisms: (i) the increased contact area of small particles within the setal ridges could result in stronger adhesion than on the antennal surface which is smoother at this length scale; ridges could also reduce contact with adjacent brush setae, and therefore avoid self-matting; (ii) electrostatic attraction of the particles could arise via contact electrification when the cleaning structure is moved across the antennal surface; or (iii) the cleaning structures could be covered by thicker layers of surface lipids than the antenna, potentially resulting in a larger contact area than on the rough antenna surface. The ants' antenna cleaner secretes surface lipids from ‘class I’ gland cells on the basitarsus [30]. It is still unknown whether these secretions indeed increase the cleaning efficiency or whether they mainly fulfil other functions such as water-proofing or anti-microbial grooming [18].

**Figure 8.**
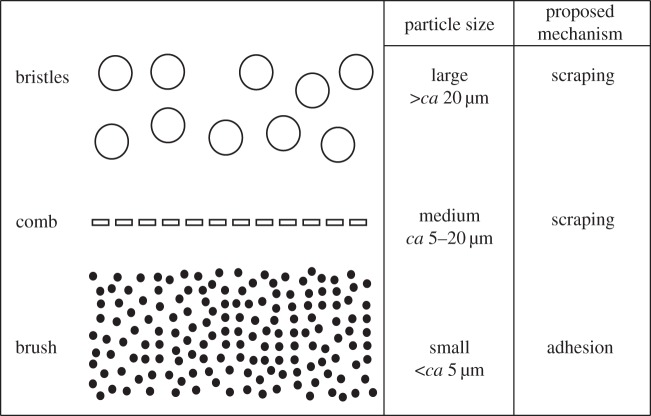
Schematic diagram summarizing how cleaning hairs on the ant's antenna cleaner may act as a sequential particle filter: (i) the bristles are responsible for picking up the largest particles from the antennae; (ii) the comb removes particles smaller than the gaps between the bristles but larger than the gaps of the comb; (iii) even smaller particles adhere to the flexible setae of the brush.

The hair arrays on the notch appear to remove particles in decreasing order of size akin to a series of consecutive sieves or filtration steps (figure 8). Most large particles will be scraped off the antenna by the bristles and the comb before they reach the brush. It is likely that large particles in the brush would impede the close contact between brush setae and antennal sensilla resulting in a less efficient removal of small particles.

The similar gap width of the combs on notch and spur (1.7 μm versus 2 μm) suggests that both fulfil a similar function. Apart from providing a filter for particles of a particular size range, the combs are probably essential for removing particles sticking to the antennal sensilla. The gap width of both combs is only slightly larger than the diameter of antennal sensilla, so that particles will be stripped off when the antenna is pulled through the cleaner.

Our high-magnification recordings of the movement of the antenna through the cleaning structure (see the electronic supplementary material) suggest that the bristles and comb teeth behave differently during the cleaning process. The bristles appear to bend when the antenna is pulled through and flick away particles when released. By contrast, the comb hairs keep tight contact with the antenna surface during cleaning. While bristles are conical, comb and brush hairs are flattened. The detailed mechanical properties of these outgrowths will need to be characterized in future work.

A better understanding of insect grooming may help to identify contaminants that insects cannot easily clean, providing an alternative way for insect pest control. For example, hairy adhesive systems of insects were found to have difficulty removing contaminants of a particular size, impeding their function [3]. Analysing the detailed biomechanics of cleaning mechanisms in insects may also help to uncover principles relevant for synthetic cleaning of surfaces at the micro- and nanoscales, such as in the fabrication of delicate semiconductor and microelectronic devices, where contamination can lead to a substantial number of defects and reduction of the manufacturing yield (e.g. [31]).

## Data accessibility

Data are available from the Dryad Repository: http://dx.doi.org/10.5061/dryad.88q18.
